# Molecularly Imprinted Polymers (MIPs) as Theranostic Systems for Sunitinib Controlled Release and Self-Monitoring in Cancer Therapy

**DOI:** 10.3390/pharmaceutics12010041

**Published:** 2020-01-03

**Authors:** Ortensia Ilaria Parisi, Mariarosa Ruffo, Rocco Malivindi, Anna Francesca Vattimo, Vincenzo Pezzi, Francesco Puoci

**Affiliations:** 1 Department of Pharmacy, Health and Nutritional Sciences, University of Calabria, 87036 Rende (CS), Italy; mariarosa.ruffo@unical.it (M.R.); rocco.malivindi@unical.it (R.M.); vincenzo.pezzi@unical.it (V.P.); 2 Macrofarm s.r.l., c/o Department of Pharmacy, Health and Nutrition Sciences, University of Calabria, 87036 Rende (CS), Italy; vattimoanna@yahoo.it

**Keywords:** Molecularly Imprinted Polymers (MIPs), molecular imprinting, theranostics, Sunitinib, cancer therapy, controlled release, Drug Delivery Systems (DDSs), adsorption and release kinetics, precipitation polymerization

## Abstract

Cytotoxic agents that are used conventionally in cancer therapy present limitations that affect their efficacy and safety profile, leading to serious adverse effects. In the aim to overcome these drawbacks, different approaches have been investigated and, among them, theranostics is attracting interest. This new field of medicine combines diagnosis with targeted therapy; therefore, the aim of this study was the preparation and characterization of Molecularly Imprinted Polymers (MIPs) selective for the anticancer drug Sunitinib (SUT) for the development of a novel theranostic system that is able to integrate the drug controlled release ability of MIPs with Rhodamine 6G as a fluorescent marker. MIPs were synthesized by precipitation polymerization and then functionalized with Rhodamine 6G by radical grafting. The obtained polymeric particles were characterized in terms of particles size and distribution, *ξ*-potential and fluorescent, and hydrophilic properties. Moreover, adsorption isotherms and kinetics and in vitro release properties were also investigated. The obtained binding data confirmed the selective recognition properties of MIP, revealing that SUT adsorption better fitted the Langmuir model, while the adsorption process followed the pseudo-first order kinetic model. Finally, the in vitro release studies highlighted the SUT controlled release behavior of MIP, which was well fitted with the Ritger-Peppas kinetic model. Therefore, the synthesized fluorescent MIP represents a promising material for the development of a theranostic platform for Sunitinib controlled release and self-monitoring in cancer therapy.

## 1. Introduction

Conventional cytotoxic agents that are used in cancer therapy are characterized by several limitations that affect their efficacy and safety profile, resulting in a high incidence of side effects, which include bone marrow suppression, nausea, vomiting, and alopecia. Anticancer drugs, indeed, present a narrow therapeutic index, lack of both selectivity and aqueous solubility, limited cell penetration, and multidrug resistance [1].

In an effort to circumvent these drawbacks and reduce the adverse reactions, many researchers have focused their attention on the development of new Drug Delivery Systems (DDSs) that are able to release the therapeutic agent in a controlled and selective way at the site of action, increasing the intracellular concentration. Many strategies have been explored and, among them, theranostics represents an innovative approach based on the integration of diagnosis and targeted therapy, which also allows the real-time monitoring of the therapeutic response [2]. Theranostic agents, indeed, are multitasking vehicles consisting of both diagnostic and therapeutic functions in the aim to achieve improved cancer therapy and tumor imaging.

Based on these considerations, the aim of this study was the preparation and characterization of Molecularly Imprinted Polymers (MIPs) that are selective for the anticancer drug Sunitinib (SUT) for the development of a novel theranostic system that is able to combine the drug controlled release ability of MIPs and Rhodamine 6G as a fluorescent marker. The use of a fluorescent material, indeed, allows the real-time imaging of cancer and thus, simultaneous diagnosis and guided therapy.

Molecular Imprinting is a very promising and powerful technology for the synthesis of crosslinked polymeric matrices, which is able to identify in a specific and selective way a target compound, called a template [3,4,5]. This effective and versatile technology involves the introduction of the template during the polymerization process and its removal after the reaction has taken place. The resulting MIPs are characterized by a permanent chemical memory for the target molecule due to the presence of complementary binding sites, which give high specificity and loading and controlled release capacities to the polymeric material. These polymers are receiving considerable attention not only for their selective recognition abilities, but also for their high stability in a wide range of conditions, including temperature, pressure and pH, reduced costs of production, and ease of preparation. Moreover, these materials can be regenerated and, therefore, reused more times without any loss in their activity. Due to their ability to control the delivery of the chosen drug used as a template during the polymerization procedure, MIPs can have applications in the development of vehicles for the sustained release of therapeutic agents characterized by a narrow therapeutic index, such as anticancer drugs.

During the last few years, MIPs with fluorescent behavior have received considerable attention due to the combination of both selective recognition abilities and fluorescent properties [6,7]. Several synthetic approaches were developed, including copolymerization of fluorescent monomers and encapsulation polymerization of nanomaterials (fluorophores) into MIPs in order to obtain fluorescent MIPs for chemo/biodetection and bio-imaging such as in vitro imaging in living cells, in vivo imaging in tissues of bodies, and bio-distribution. In a recent study, fluorescent molecularly imprinted nanoparticles were prepared for active targeted drug delivery and imaging using N-terminal epitope of P32 membrane protein and doxorubicin as templates to actively recognize the P32-positive 4T1 cancer cells [8].

In this work, a MIP selective for Sunitinib was prepared by precipitation polymerization and was then functionalized with Rhodamine 6G by a radical grafting procedure. SUT, indeed, represents one of the most widely used tyrosine kinase inhibitors (TKI) for the treatment of several kinds of cancer [9,10]. This therapeutic agent has been approved by the US Food and Drug Administration (FDA) for the management of gastrointestinal stromal tumor (GIST), pancreatic neuroendocrine tumor (PNT), and renal-cell carcinoma (RCC). In particular, the SUT mechanism of action involves the inhibition of vascular endothelial growth factor receptor (VEGFR-1,2 and 3), platelet-derived growth factor receptor (PDGFR-α and β), stem cell factor receptor (KIT), neurotrophic factor receptor, Fms-like TK-3 (FLT3), and colony stimulating factor receptor type 1. Some of these receptors are expressed in different malignancies; therefore, SUT can also have applications in other antitumor therapies. However, this compound is responsible for the onset of important side effects such as arterial thrombosis, hemorrhage, hypothyroidism, congestive heart failure, wound dehiscence, and renal dysfunction [11,12,13,14,15,16]. Moreover, due to its poor water solubility, dissolution rate, and oral bioavailability, many researchers have explored different strategies to prepare devices that are able to overcome these limitations. A series of novel multi-block copolymers consisting of poly(l-lactide) blocks (PLLA) and blocks containing poly(d,l-lactide) (PDLLA) and polyethylene glycol (PEG) (PDLLA-PEG-PDLLA) were prepared and employed to produce sunitinib-loaded polymeric microspheres as intravitreal formulation for the treatment of ocular diseases [17]. In another study, SUT was encapsulated in alginate nanoparticles using calcium chloride as a crosslinker [18], while Alshetaili et al. developed three different formulations for SUT delivery to colon cancer cells by the nanoprecipitation technique using various concentrations of poly-lactic-*co*-glycolic acid (PLGA) and pluronic acid as surfactant [19]. Another interesting strategy involved the synthesis of a sunitinib-sericin conjugate by simple click reaction in water using L-ascorbic acid/hydrogen peroxide as a free radical grafting initiator [20]. On the contrary, only two works report on SUT imprinted polymers [4,5]. The first one was focused on the synthesis of a MIP based on methacrylic acid (MAA) and ethylene glycol dimethacrylate (EGDMA) by bulk polymerization according to the non-covalent approach. Rebinding experiments confirmed the ability of the prepared MIP to bind in a specific and selective fashion the template molecule. In the second study, several fluorescent imprinted nanogels for SUT detection in human plasma were synthesized using a selection of functional monomers based on different aminoacids and coumarin. In particular, the fluorescence quenching of the coumarin-based nanogel allowed the direct detection of the anticancer drug in human plasma.

Here, a new fluorescent SUT imprinted polymer was prepared by precipitation polymerization, using MAA and EGDMA as a monomer and crosslinker, respectively, followed by the grafting of Rhodamine 6G. The obtained MIP was characterized in terms of adsorption isotherms and kinetics and in vitro release properties. The obtained results confirmed that the prepared imprinted polymer could represent a promising material for the development of a theranostic platform that is able to combine the drug controlled release ability of MIPs with Rhodamine 6G as a fluorescent marker and, therefore, with the real-time fluorescent imaging of cancer for simultaneous diagnosis and guided therapy.

## 2. Materials and Methods

### 2.1. Materials

Methacrylic acid (MAA), ethylene glycol dimethacrylate (EGDMA), 2,2′-azobisisobutyronitrile (AIBN), sunitinib malate (SUT), semaxanib (SEM), rhodamine 6G (R6G), L-ascorbic acid (AA), hydrogen peroxide, bovine serum albumin (BSA), disodium hydrogen phosphate (Na_2_HPO_4_), and sodium dihydrogen phosphate (NaH_2_PO_4_) were purchased from Sigma-Aldrich s.r.l. (Milan, Italy).

Methacrylic acid was purified before use on an alumina column by a single-step passage, while AIBN was recrystallized from methanol to give colorless needles.

All solvents were reagent or HPLC grade and obtained from VWR (Milan, Italy).

Dialysis membranes (molecular weight cut-off, MWCO: 3500 Da) were used for the in vitro release studies and were supplied by Medicell International Ltd. (London, UK).

### 2.2. Instrumentation

Absorption spectra were recorded with a Jasco V-530 UV/Vis spectrometer (JASCO International Co., Ltd. Tokyo, Japan).

Particles size and distribution were evaluated by Dynamic Light Scattering (DLS) employing a 90 Plus Particle Size Analyzer (Brookhaven Instruments Corporation, New York, NY USA) at 25.0 ± 0.1 °C. The autocorrelation function was measured at 90° and the laser was operating at 658 nm. The size distribution was obtained from the instrumental data fitting by the inverse “Laplace transformation” and Contin methods [21]. The polydispersity index (PI) was used as a measure of the size distribution and a value less than 0.3 indicates a homogeneous population of particles.

The *ξ*-potential was measured using a Zetasizer ZS (Malvern Instruments Ltd., Malvern, UK) at 25.0 ± 0.1 °C. *ξ*-potential values were calculated by the instrument software using the Helmholtz–Smoluchosky equation.

For particle size and *ξ*-potential measurements, 25 μL of a polymeric particles suspension (1 mg/mL) in phosphate buffer solution at pH 7.4 were added to 3 mL of PBS and sonicated for 30 s.

All the analyses were repeated in triplicate and expressed as mean ± standard deviation.

Particles morphology and fluorescence were investigated by fluorescence microscopy. The fluorescence images of the prepared particles were obtained with an Olympus BX41 microscope equipped with an Olympus XC30 camera (Olympus Optical Co. (UK) Ltd, London, UK). Data acquisition and elaboration were performed with the Olympus cellSens Imaging Software Version 1.11.

pH-modulated fluorescence was investigated using a Synergy H1 spectrofluorometer (Hybrid Reader, BioTek, Winooski, VT, USA). The emission spectra (excitation at *λ*_ex_ = 510 nm) of the fluorescent MIP as a function of the media pH were recorded using an aqueous dispersion of the polymeric particles (0.5 mg/mL) at different pH values.

### 2.3. Synthesis of the Sunitinib Molecularly Imprinted Polymer (SUT-MIP)

The Molecularly Imprinted Polymer selective for Sunitinib was synthesized by precipitation polymerization using methacrylic acid (MAA), ethylene glycol dimethacrylate (EGDMA), and 2,2′-azoisobutyronitrile (AIBN) as a functional monomer, crosslinker, and radical initiator, respectively.

In a 100 mL round-bottom flask, 100 mg (0.25 mmol) of the template molecule and 0.68 mL (8 mmol) of MAA were dissolved in 60 mL of a methanol/acetonitrile mixture (1:1 *v*/*v*). The obtained solution was sonicated for 10 min in the aim to promote the formation of the template-functional monomer complex. Then, 1.89 mL (10 mmol) of EGDMA and 100 mg of AIBN were added and the reaction mixture was sonicated for another 10 min and purged with nitrogen. The flask was rotated at 40 rpm during the polymerization reaction, which was carried out at 60 °C. After 24 h, the obtained polymeric particles were recovered by filtration and washed with dimethyl sulfoxide (DMSO), acetone, and, finally, diethyl ether.

The template removal was performed using a Soxhlet apparatus. For this purpose, an acetic acid-methanol mixture (1:9 *v*/*v*) was employed as an extraction solvent for the first 48 h, followed by methanol for another 48 h. At the end, the polymeric particles were dried under vacuum overnight at 40 °C.

MIP was checked to be free of SUT and any other compound by UV-Vis analysis.

A Non-Imprinted Polymer (NIP) was also synthesized according to the same experimental protocol adopted for the imprinted particles, but in the absence of the drug.

### 2.4. Functionalization of Imprinted and Non-Imprinted Polymeric Particles

The synthesized polymeric beads were functionalized with the fluorescent dye Rhodamine 6G (R6G) according to the following radical grafting procedure.

Briefly, 50 mg of Rhodamine 6G was dissolved in 50 mL of distilled water and, then, 1.0 g of polymeric particles were added. The obtained polymeric suspension was maintained under magnetic stirring at 25 °C. The grafting reaction was started by adding 2.5 mL of H_2_O_2_ (30% *v*/*v*) containing 83.5 mg of L-ascorbic acid. After 24 h, the functionalized polymeric materials were recovered by filtration, washed with methanol, acetone, and diethyl ether and, finally, dried under vacuum overnight at 40 °C.

### 2.5. Binding Studies

#### 2.5.1. Static Equilibrium Adsorption Experiments

Binding studies were carried out with the aim to investigate both the recognition properties and the selectivity of synthesized MIPs.

The experiments were performed in a methanol/acetonitrile mixture (25:75 *v*/*v*) using 100 mg of the imprinted and non-imprinted particles, which were mixed with 2 mL of a Sunitinib standard solution (1.0 × 10^−5^–1.0 × 10^−3^ M). After 24 h, each sample was centrifuged at 9000 rpm for 10 min and the SUT concentration was determined by UV-Vis analysis at 421 nm using the equation obtained from the calibration curve of the drug.

In the aim to investigate the selectivity of MIP, non-competitive binding studies were also carried out in the presence of Semaxanib (SEM), which is a structural homologue of SUT, following the same experimental procedure.

The binding experiments were repeated in triplicate.

#### 2.5.2. Kinetic Adsorption Experiments

Adsorption kinetic studies of the synthesized particles were carried out as described below.

100 mg of polymeric beads were mixed with 2 mL of a SUT standard solution (4.0 × 10^−4^ M) in a methanol/acetonitrile mixture (25:75 *v*/*v*). The drug concentration was monitored by UV-Vis analysis at different incubation times (1–24 h), after which samples were centrifuged at 9000 rpm for 10 min.

SUT concentration was determined at 421 nm using the equation obtained from the calibration curve of the therapeutic agent.

The experiments were performed in triplicate.

### 2.6. Protein Adsorption Measurement and Swelling Behavior

Protein adsorption experiments and swelling studies were carried out according to the experimental protocols reported in a previous work [22].

For the protein adsorption measurement, a BSA standard solution (1.2 mg/mL) was prepared in phosphate buffer at pH 7.4 (25 mM). Then, 150 mg of MIP and NIP particles were packed into 6.0 mL polypropylene SPE columns, which were preconditioned by successive washing steps with water, HCl (0.07 M), water, CH_3_OH/water (50:50, *v*/*v*), water, and phosphate buffer (25 mM, pH 7.4). Each cartridge was loaded with 2.0 mL of BSA standard solution and the amount of adsorbed protein was calculated by using a UV-Vis spectrophotometer at 290 nm. Experiments were repeated in triplicate.

For the swelling studies, 50 mg of MIP and NIP particles were placed into a tared 5 mL sintered glass filter (Ø 10 mm; porosity, G3), weighed, and left to swell in phosphate buffer at pH 7.4. After 24 h, the excess water was removed by percolation and, then, the filter was centrifuged at 3500 rpm for 15 min and weighed. The filter tare was measured after centrifugation with only water. The water content percentage (*WR*%) was calculated according to Equation (1):

(1) $$WR\%=\frac{W_{s}-W_{d}}{W_{d}}\times 100$$

where *W*_s_ and *W*_d_ are weights of swollen and dried particles, respectively. Each experiment was carried out three times.

### 2.7. Drug Loading Procedure and In Vitro Release Studies

In a 10 mL round-bottom flask, 10 mg of SUT were dissolved in 3 mL of an ethanol/DMSO mixture (2:1 *v*/*v*) and sonicated for 10 min. Then, 90 mg of MIP and NIP were introduced and samples were soaked under continuous stirring in dark conditions at room temperature. After three days, the polymeric particles were transferred into sintered glass filters to remove the supernatant by percolation and, finally, dried under vacuum at 40 °C overnight.

The SUT equilibrium concentration and, therefore, the amount of unloaded drug was quantified by analyzing the obtained leachate by UV-Vis spectroscopy.

In this way, Drug Loading Content (DLC) and Drug Loading Efficiency (DLE) were determined according to Equations (2) and (3), respectively:

(2) $$DLC\;\left(\%\right)=\;\frac{weight\;of\;loaded\;SUT}{\left(weight\;of\;loaded\;SUT+\;weight\;of\;loaded\;polymeric\;particles\right)}\;\times 100$$

(3) $$LE\;\left(\%\right)=\;\frac{weight\;of\;loaded\;SUT}{weight\;of\;SUT\;used\;in\;loading\;procedure}\times 100$$

In vitro release studies were carried out in phosphate buffer at pH 7.4 (10^−3^ M) according to the dialysis bag diffusion technique [23]. For this purpose, 20 mg of SUT loaded particles were immersed in 1 mL of PBS and placed in a dialysis tube, which was sealed at each end with clamps. Then, the tube was immersed in a flask containing 10 mL of phosphate buffer and incubated at 37 ± 0.5 °C for 24 h. At selected time intervals (0.5, 1, 1.5, 2, 2.5, 3, 4, 6, 8, 10, 12, and 24 h), 3 mL of sample was withdrawn from the medium and was then restored to the initial volume with fresh PBS. SUT concentration was quantified by UV-Vis analyses and the obtained percentages of released therapeutic agent were used to characterize the release profile.

Experiments were repeated three times.

## 3. Results and Discussion

### 3.1. Synthesis of Sunitinib Molecularly Imprinted Polymers Functionalized with Rhodamine 6G (R6G)

Fluorescent MIP particles selective for Sunitinib were synthesized by precipitation polymerization using methacrylic acid (MAA), ethylene glycol dimethacrylate (EGDMA), and 2,2′-azoisobutyronitrile (AIBN) as a functional monomer, crosslinking agent, and radical initiator, respectively, and according to the non-covalent imprinting approach.

**Figure 1:**
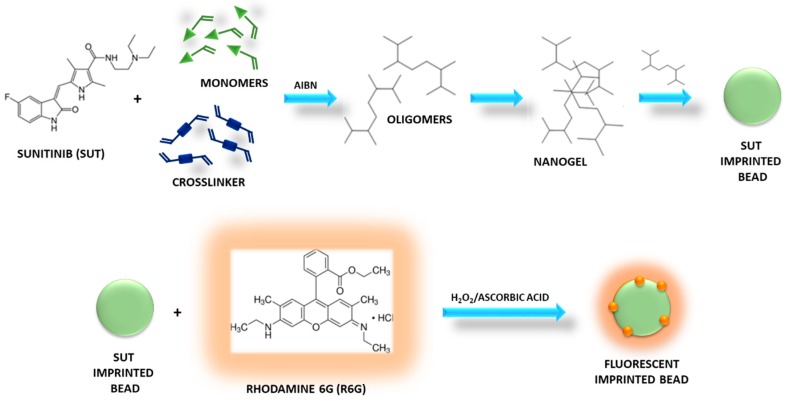
Synthesis of Sunitinib (SUT) imprinted beads grafted with Rhodamine 6G.

In the precipitation polymerization, as the reaction starts, monomers form oligomer radicals and then, the formed oligomers crosslink, leading to crosslinked nuclei. The oligomers are still soluble in the reaction solvent, while the nuclei grow capturing monomers and oligomers until they reach a critical mass and precipitate, forming polymeric beads (Figure 1) [24].

This synthetic technique is characterized by several advantages such as easy preparation, absence of stabilizers and other additives, compatibility with high amounts of crosslinker, and use of aprotic solvents as porogen, which are able to promote non-covalent interactions between template and monomer.

In the present research study, indeed, the non-covalent approach was selected to prepare SUT imprinted particles due to its characteristics that make it the most widely used method for the production of MIPs. This method involves the formation of reversible non-covalent interactions (hydrogen bonds, van der Waals forces, ion pair, and dipole–dipole interactions) between the target molecule and the selected functional monomers during both the polymerization reaction and the rebinding phase. It consists of a simple experimental procedure for the formation of the template-monomer pre-polymerization complex and the template extraction.

Methacrylic acid was chosen as a functional monomer due to its ability to form reversible hydrogen bonds with the drug molecule, while EGDMA provided mechanical stability to the polymeric material and stabilized monomer functionalities around the template molecule and, thus, the architecture of the recognition cavities after the template extraction. Moreover, MAA and EGDMA are both biocompatible in their polymerized form and their wide use in the preparation of polymers for pharmaceutical and biomedical applications is extensively reported in literature [25,26,27,28].

A template/monomer/crosslinker molar ratio equal to 0.25:8:10 was employed according to our previous experience and the obtained experimental results.

After polymerization, the recovered particles were functionalized by radical grafting of R6G in heterogeneous phase. For this purpose, H_2_O_2_/L-ascorbic acid redox pair was used as a biocompatible and water-soluble initiating system [29]. L-ascorbic acid is characterized by two ionizable hydroxyl groups. The dissociation of 3-OH and 2-OH produces ascorbate monoanion (AscH^−^) and ascorbate dianion (Asc^2−^). The first one is a very good reducing agent, which undergoes two consecutive one-electron oxidation reactions, leading to the formation of ascorbate radical (Asc^•−^) and, then, dehydroascorbic acid (DHA) [30].

Ascorbic acid undergoes autoxidation in aqueous solution, generating ascorbate radicals, but the presence of hydrogen peroxide accelerates the reaction, of which its mechanism is reported below:

AA + H_2_O_2_ → Asc^•−^ + 2H_2_O

Therefore, the ascorbate radicals generated by the redox pair react with the preformed polymers, abstracting carbon-bound hydrogens to form macroradicals. There are, indeed, two sites for radical attack, such as methyl and methylene groups. Then, R6G reacts with the obtained macroradicals and is grafted onto the polymeric particles, which were collected after washing and drying.

### 3.2. Characterization of Fluorescent Sunitinib Molecularly Imprinted Polymer (SUT-MIP)

**Figure 2:**
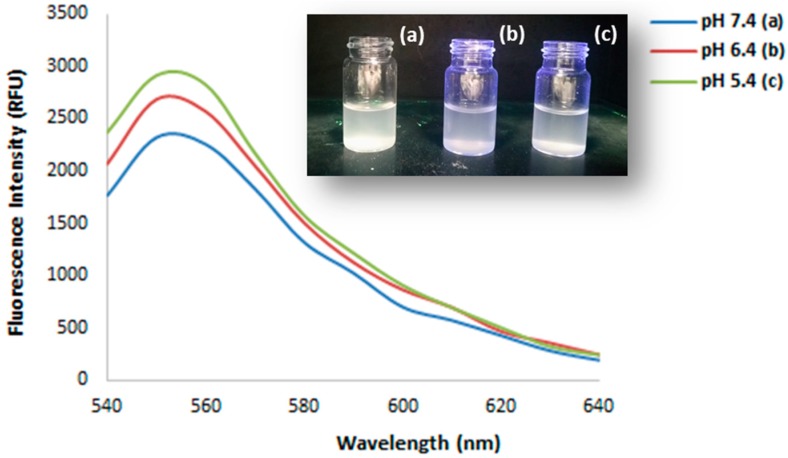
Fluorescence emission spectra of Molecularly Imprinted Polymers (MIP) as a function of the media pH and particles dispersions at pH 7.4 (**a**), 6.4 (**b**), and 5.4 (**c**) under 254 nm UV light.

Fluorimetric characterization of the polymer was carried out, recording the emission spectra of particles dispersions in aqueous solution at different pH (Figure 2) [31].

The dispersion at pH 5.4 showed a higher fluorescence emission compared to the samples at pH 6.4 and 7.4. These results are ascribable to the pH-sensitivity of Rhodamine 6G (R6G), which is a fluorochrome that is widely employed in the theranostic field. This molecule, indeed, presents a strong fluorescence only in acidic environments and this feature plays a key role in the real-time tumor imaging due to the pH difference between tumor and normal cells [32]. Therefore, the functionalization of the synthesized MIP particles with R6G makes the prepared polymer a promising material for the development of a theranostic system for cancer imaging-guided diagnosis and therapy.

The efficiency of the grafting reaction was investigated, dosing the remaining dye molecules in solution after washing and recovery of the polymeric particles by filtration. For this purpose, R6G reference solutions were prepared to record a calibration curve of the dye. Then, the fluorescence of the supernatant was measured, gaining a signal that was ascribable to the not reacted rhodamine and the amount of bound fluorescent marker was expressed as mg equivalent of R6G per gram of polymer (mg eq R6G/g). Based on the obtained spectra, this value was equal to 3.7 ± 0.6 mg eq R6G/g.

**Figure 3:**
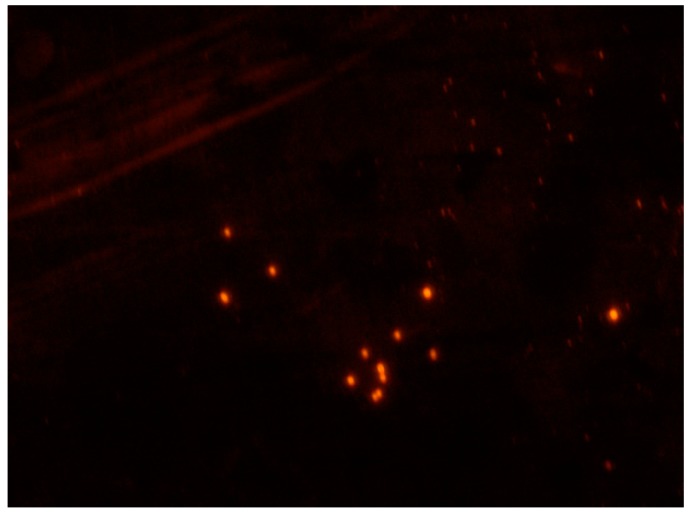
Fluorescence microscopy image (magnification 40x) of SUT imprinted particles grafted with Rhodamine 6G (R6G).

Moreover, fluorescence microscopy images of SUT imprinted beads grafted with R6G are shown in Figure 3.

Particles size and size distribution were evaluated by Dynamic Light Scattering (DLS) and no significant differences were observed between imprinted particles and the corresponding non-imprinted ones. The mean diameter is around 700 nm with an acceptable polydispersity index (Table 1.). Moreover, in both the cases, particle surfaces are negatively charged due to the deprotonation of MAA acid groups at physiological pH (7.4). *ξ*-potential values, indeed, were equal to −23.8 ± 0.3 and −29.3 ± 0.4 mV for MIP and NIP particles, respectively, indicative of a certain electrostatic repulsive force between the polymeric particles, which provides a significant stability.

### 3.3. Binding Studies

#### 3.3.1. Imprinting Efficacy and Selectivity

The study of the adsorption isotherms allows one to describe the nonlinear and dynamic equilibrium between the amount of template adsorbed on the polymeric matrix and the amount of template in the solution. Moreover, the analysis of the isotherm data represents a crucial point in the optimization of the use of Molecularly Imprinted Polymers.

An adsorption isotherm, indeed, is a curve relating the amount of an analyte, in this case represented by the template molecule, adsorbed onto the polymer at equilibrium (*Q*_e_) and the equilibrium concentration of the same analyte in solution (*C*_e_). The *Q*_e_/*C*_e_ relationship depends on the type of adsorption process that occurs. Therefore, adsorption isotherms, which can take multiple forms depending on the adopted model, allow one to acquire useful information concerning the template–polymer interaction.

In order to evaluate the adsorption properties of the synthesized polymers, SUT imprinted particles and the corresponding non-imprinted ones were incubated for 24 h with SUT standard solutions at different concentrations prepared in a methanol/acetonitrile mixture (25:75 *v*/*v*).

The performed binding experiments allowed us to determine the binding capacity of MIP and NIP particles, which represents the amount of SUT bound to the polymers at equilibrium (*Q*_e_, mol/g) according to Equation (4) [33]:

(4) $$Q_{e}=\frac{\left(C_{i}-C_{e}\right)\times V}{m}$$

where *C*_i_ and *C*_e_ (mol/L) are the initial and equilibrium SUT concentrations in the solution, respectively, *V* (L) is the volume of the solution, and *m* (g) is the weight of the polymers.

**Figure 4:**
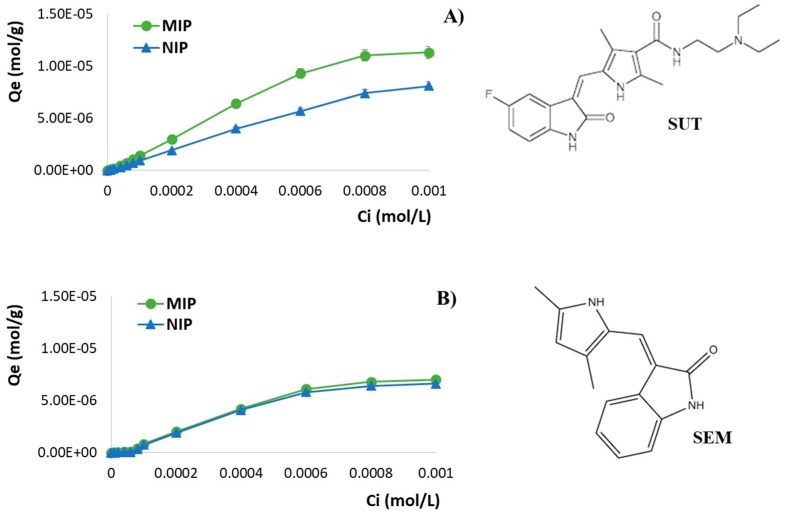
Adsorption isotherms of (**A**) SUT and (**B**) SEM on imprinted and non-imprinted particles and chemical structures of Sunitinib (SUT) and Semaxanib (SEM).

By plotting *Q*_e_ versus *C*_i_, the adsorption isotherms of Sunitinib on MIP and NIP particles were obtained (Figure 4A).

In the present research study, the obtained results confirmed the ability of the imprinted particles to bind a higher amount of drug compared to the corresponding non-imprinted ones. Moreover, with the increase of the SUT initial concentration *C*_i_, the adsorption capacities of both the polymeric materials also increased until a saturation point.

The same experiments were also carried out using Semaxanib (SEM) standard solutions at different concentrations in order to perform non-competitive binding studies and to investigate the cross-reactivity of MIP in the presence of a structural analogue of SUT (Figure 4B). As reported in Table 2, the percentages of bound SUT and SEM confirm the imprinting efficacy and the selectivity of the prepared imprinted particles.

The relevant molecular recognition properties for SUT exhibited by the prepared fluorescent MIP are ascribable to selective binding cavities, which are template-complementary in size, shape, and chemical functionalities. These binding sites which exist in the imprinted polymers were formed during the polymerization, which was carried out in the presence of the target therapeutic agent. On the other hand, non-specific interactions are responsible for the bound amount of SEM for both the polymeric matrices.

The imprinting factor (*α*) and the selectivity coefficient (*ε*) were also determined for the synthesized polymeric materials and the obtained results are shown in Table 2. The first parameter represents the ratio between the amount of analyte (template or its analogue) adsorbed by MIP and the amount of analyte adsorbed by NIP, while the second parameter is calculated as the ratio between the amount of SUT and the amount of SEM adsorbed by MIP. *α* and *ε* values confirmed the selective recognition abilities for the therapeutic agent of the imprinted material compared to the corresponding non-imprinted one. The obtained *α* SUT values are higher than 1.20 for each adopted *C*_i_, which is indicative of the MIP better ability to bind the therapeutic agent compared to the corresponding non-imprinted polymer. In addition, the imprinted particles are from 1.49 to 7.37 times more selective for SUT according to the observed *ε* values.

**Figure 5:**
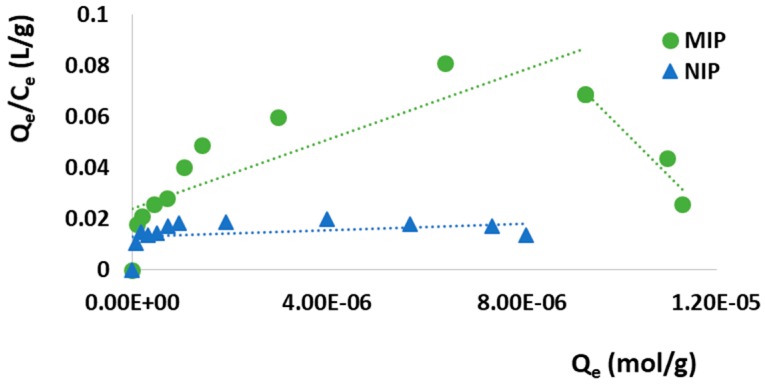
Scatchard analysis.

In the aim to better study the adsorption properties of the synthesized MIP and the affinity distribution of the recognition sites, the obtained binding data were plotted according to the Scatchard model (Equation (5)), which allows one to discriminate between homogeneous and heterogeneous sites:

(5) $$\frac{Q_{e}}{C_{e}}=\left(B_{\max}-Q_{e}\right)K_{a}$$

where *Q*_e_ is the amount of SUT bound per gram of polymeric material at the equilibrium (mol/g), *C*_e_ is the drug equilibrium concentration (mol/L), *B*_max_ (mM/g) is the apparent maximum binding capacity, and *K*_a_ (M^−1^) is the association constant. Therefore, by plotting *Q*_e_/*C*_e_ versus *Q*_e_, the binding parameters, such as *K*_a_ and *B*_max_, were estimated from the slope and the intercept, respectively (Figure 5 and Table 3).

As it is possible to observe in Figure 5, the relationship between *Q*_e_/*C*_e_ and *Q*_e_ of MIP is not linear, but consists of two different straight lines with different slopes suggesting that the imprinted particles are characterized by heterogeneous binding cavities, which can thus be distinguished into high-affinity and low-affinity sites. On the contrary, the NIP Scatchard plot is composed of a single line, indicating the presence of only one type of binding site.

The collected experimental binding data were also fitted using the Langmuir and Freundlich models and their linearization.

The Langmuir model allows one to determine the maximum adsorption capacity (*Q*_max_) and it is provided by the following Equation (6):

(6) $$\frac{1}{Q_{e}}=\frac{1}{Q_{\max}C_{e}K_{L}}+\frac{1}{Q_{\max}}$$

where *Q*_e_ is the amount of SUT bound per gram of polymeric particles at the equilibrium (mol/g), *C*_e_ is the drug equilibrium concentration (mol/L), *Q*_max_ is the maximum adsorption capacity corresponding to complete monolayer coverage on the surface (mol/g), and *K*_L_ is the Langmuir constant related to the energy or net enthalpy of sorption (L/mol). The plot of 1/*Q*_e_ versus 1/*C*_e_ gave two straight lines and *K*_L_ and *Q*_max_ for the two synthesized polymeric materials were calculated from the slope and the intercept, respectively (Table 4).

The Langmuir model takes into account a monolayer coverage and, thus, an adsorption process on a homogeneous polymeric surface consisting of a restricted number of uniform recognition sites. Each site is able to bind just one molecule; therefore, once a site is occupied, no additional adsorption can occur at the same site. The maximum adsorption capacity is achieved when polymeric surface reaches saturation. Moreover, this model provides that the ability of the target analyte to bind a specific site is not influenced by the occupation of the close sites. As shown in Table 4, NIP *Q*_max_ is higher than the value observed for the corresponding MIP. This model, indeed, assumes that all the recognition sites are homogeneous, thus, the obtained *Q*_max_ values can be acceptable. However, Molecularly Imprinted Polymers do not always fit so well to a homogeneous model.

The Freundlich isotherm is introduced as an empirical model, which involves a non-ideal adsorption process on heterogeneous surfaces assuming that as the analyte concentration increases, the concentration of the analyte on the adsorbent surface will increase. Contrary to the Langmuir model described above, this one is not limited to the formation of a monolayer, but it assumes that the polymer surface recognition holes are not evenly distributed and the adsorption might occur in the multimolecular layer [34]. Freundlich isotherm is explained by the following Equation (7) and its linear form (8):

(7) $$Q_{e}=K_{F}C_{e}{}^{m}$$

(8) $$logQ_{e}=logK_{F}+mlogC_{e}$$

where *K*_F_ is the Freundlich constant related to the binding affinity ((mol/g)(mol/L)^m^), which represents an indicator of the adsorption capacity, and m is the heterogeneity index (dimensionless), which ranges from 0 (for a heterogeneous system) to 1 (for a homogeneous system). By plotting log*Q*_e_ versus log*C*_e_, *K*_F_ and m values, which are system specific parameters, can be determined (Table 4). In the present study, Freundlich isotherm shows a good linearity for SUT adsorption on both imprinted and non-imprinted polymers (*R*^2^ equal to 0.93 and 0.99, respectively). Moreover, both the synthesized polymeric materials are characterized by m values above 1, which is indicative of a cooperative adsorption. This kind of process occurs when the binding of one molecule affects the binding of others [35].

Based on the obtained *R*^2^ values, which were shown in Table 4, SUT adsorption on the prepared MIP better fit the Langmuir model. According to this isotherm model, the adsorption process involved definite homogeneous sites, resulting in a monolayer coverage of the drug at the polymer surface.

#### 3.3.2. Adsorption Kinetics

Adsorption kinetics consists of the measure of the adsorption uptake with respect to time at a constant drug concentration. In the present work, adsorption kinetics was studied immersing 100 mg of the polymeric particles in 2 mL of a Sunitinib standard solution (4.0 × 10^−4^ M) and monitoring the drug concentration at different time intervals.

The amount of therapeutic agent bound at time *t* (*Q*_t_, mol/g) was determined as the difference between the initial SUT concentration at *t* = 0 (*C*_i_, mol/L) and the residual concentration at the adsorption time t (*C*_t_, mol/L) according to Equation (9):

(9) $$Q_{t}=\left(C_{i}-C_{t}\right)\frac{V}{m}$$

where *V* (L) is the volume of the incubation solution and *m* (g) is the amount of polymeric material.

**Figure 6:**
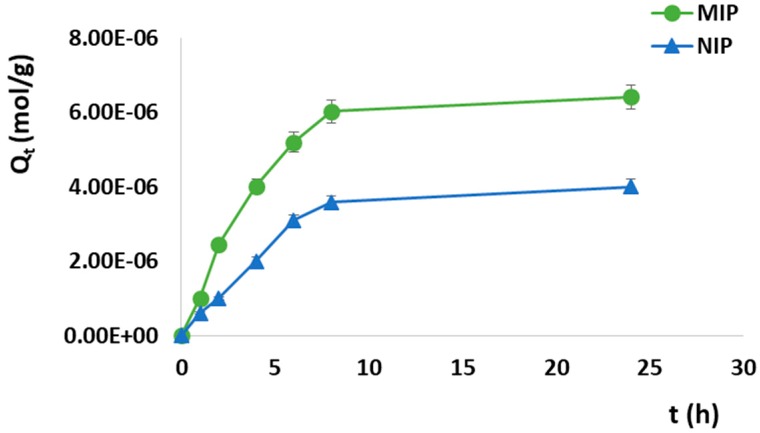
SUT adsorption kinetic curves for MIP and Non-Imprinted Polymer (NIP).

The adsorption kinetic curves for the synthesized imprinted and non-imprinted particles were shown in Figure 6 by plotting *Q*_t_ versus *t*.

The adsorption process proceeded quickly within the first 8 h and then, it became slower during the subsequent phase until it reached equilibrium and a plateau phase was observed. Furthermore, the adsorption abilities of non-imprinted particles were lower at every time point than that of the corresponding imprinted particles. After the first hour, indeed, 12.5% of SUT was adsorbed by MIP, getting 50.1%, 65.0%, 75.3%, and 80.3% at the time points of 4, 6, 8, and 24 h, respectively. On the other hand, the amount of bound Sunitinib within the first hour was equal to 7.5% for the corresponding non-imprinted particles, reaching 25.0%, 38.7%, 44.8%, and 50.0% at the time point of 4, 6, 8, and 24 h, respectively.

Kinetic models of pseudo-first order and pseudo-second order were fitted with the obtained experimental data in order to study the kinetics of SUT adsorption on the prepared MIP.

The pseudo-first order or Lagergren model is expressed by the following Equation (10):

(10) $$log\left(Q_{e}-Q_{t}\right)=log\left(Q_{e}\right)-\frac{K_{1}}{2.303}t$$

in which *Q*_e_ is the amount of adsorbed SUT at the equilibrium time, *K*_1_ represents the first-order adsorption rate constant, and t is the adsorption time. This model is based on the assumption that one molecule adsorbs within the active site of the polymer [36] and that the drug adsorption process controlled by the diffusion step belongs to the physical adsorption [34].

The pseudo-second order model is described by Equation (11):

(11) $$\frac{t}{Q_{t}}=\frac{1}{K_{2}Q_{e}{}^{2}}+\frac{1}{Q_{e}}t$$

where *K*_2_ is the pseudo-second order adsorption rate constant.

This kinetic model assumes that one adsorbate molecule interacts with two active sites [36] and that the adsorption process is a chemical process, which could be the rate-limiting step [34].

The kinetic fitting data for the imprinted and non-imprinted particles were reported in Table 5.

The applicability of the two considered kinetic models to the adsorption behavior of MIP and NIP was analyzed taking into account the obtained correlation coefficients (*R*^2^) as well as the experimental and calculated binding capacity (*Q*_e_). Based on these data, the adsorption process followed a pseudo-first order kinetics for both the imprinted and non-imprinted materials, due to the better fit of *R*^2^ values. Moreover, the theoretical *Q*_e_ values estimated from the pseudo-first order kinetic model were closer to the experimental ones than *Q*_e_ values obtained from the pseudo-second order equation, which resulted in not being suitable for the adopted experimental conditions. Furthermore, the higher MIP *K*_1_ value suggested that the adsorption rate of the imprinted particles was apparently greater than that of the corresponding non-imprinted particles.

### 3.4. Protein Adsorption Measurement and Swelling Behavior

The optimization of a drug delivery system requires a deeper understanding of the processes occurring at the interface between the developed material and the biological matrix and its components such as proteins and lipids. These processes are affected by the hydrophilic properties of the material, which can be investigated by performing protein adsorption experiments and swelling studies.

In medical applications, the interaction of a polymeric material with the biological matrix, such as the unspecific protein adsorption on the polymer surface, could affect both the efficacy and the biocompatibility of the prepared material interfering with the drug release process and the release kinetics. Therefore, protein adsorption studies were carried out using Bovine Serum Albumin (BSA), which is a physiologically relevant protein which is ubiquitous in human serum, as reference. The BSA binding capacity of the imprinted and non-imprinted particles was equal to 45.4 ± 0.2% and 32.7%, respectively, confirming an unspecific adsorption compatible with biological applications.

Swelling studies were performed with the aim to determine the water uptake capacity of the prepared polymeric matrices and, therefore, to evaluate the hydrophilic properties. For this purpose, the prepared particles were immersed in PBS at pH 7.4 for 24 h, obtaining a water uptake (%) equal to 376.1 ± 0.2 and 384.3 ± 0.3 for the imprinted and non-imprinted polymers, respectively. The obtained results highlighted good swelling properties, which allow the drug molecule to more easily access the binding cavities. The accessibility of the recognition holes to the template affects the effectiveness of MIPs; thus, a better accessibility in PBS can potentially also improve the binding abilities of the polymeric material in this aqueous-based medium.

### 3.5. In Vitro Release Studies

In the aim to investigate the specific release characteristics of the prepared particles, the cumulative drug release from MIP and NIP was determined.

For this purpose, both imprinted and non-imprinted particles were loaded by soaking them into a SUT standard solution for three days. The drug loading process, indeed, consists of physical diffusion of the therapeutic agent molecules into the two prepared polymeric materials, which involves the formation of interactions such as hydrogen bonds and non-covalent and electrostatic interactions. The type of interactions established between template and polymers is due to the exposed functional groups.

After the soaking procedure, the Drug Loading Content (DLC) and the Drug Loading Efficiency (DLE) were determined according to Equations (2) and (3) and the obtained values are shown in Table 6.

These values further highlight the selective recognition properties of the synthesized imprinted particles compared to the corresponding non-imprinted ones.

**Figure 7:**
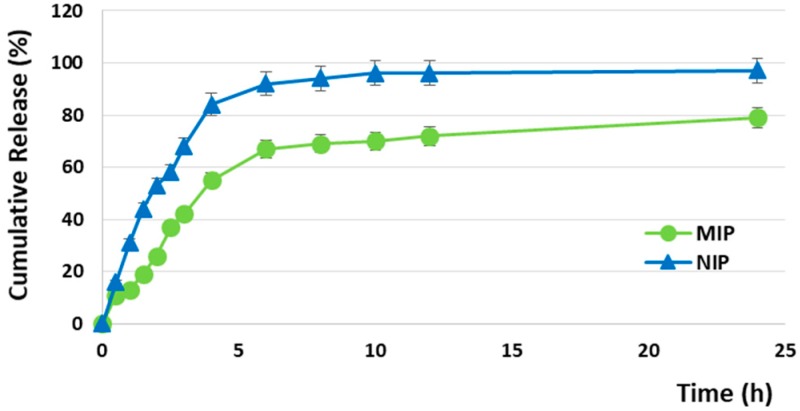
Cumulative release profiles of Sunitinib from MIP and NIP.

The Sunitinib in vitro release profiles from MIP and NIP particles are reported in Figure 7 and, as it is possible to observe, the release process consists of three main phases, namely burst, time delay, and stable release.

NIP particles exhibited a burst release equal to 53% within the first two hours, reaching the 92% and 97% in 6 and 24 h, respectively. On the contrary, MIP particles released 26% after the first two hours, achieving 67% and 79% at the 6 and 24 h time points. The observed drug release behavior from MIP is ascribable to two main factors, namely the diffusion process and the imprinting effect, due to the presence of selective recognition sites within the polymer, which are able to interact strongly with SUT molecules.

In order to explore the kinetics of the in vitro drug release and to understand the mechanism of the release process from the prepared device, different kinetic models, such as zero-order, first-order, Higuchi, and Ritger-Peppas, were applied according to the following Equations (12–15, respectively) [23]:

(12) $$\frac{M_{t}}{M_{\infty }}=K_{0}t$$

(13) $$\ln\left(1-\frac{M_{t}}{M_{\infty }}\right)=-K_{1}t$$

(14) MtM∞=KHt12

(15) $$\frac{M_{t}}{M_{\infty }}=K_{p}t^{n}$$

where *M*_t_ is the cumulative amount of drug released at time point t, *M*_∞_ is the total amount of drug incorporated into the device, *K*_0_, *K*_1_, *K*_H_, and *K*_p_ are the zero-order, first-order, Higuchi, and Ritger-Peppas rate constants, respectively, and n is the diffusional release exponent, which is indicative of the operating release mechanism [37].

Equation (14) is used successfully for the analysis of the first 60% of the release profiles [38].

As reported in Table 7, the Ritger-Peppas kinetic model provides the best fit for both MIP and NIP due to the higher *R*^2^; in addition, the observed *n* values for MIP and NIP are <0.85, indicating an anomalous (non-Fickian) transport in which both drug diffusion and polymer relaxation control the overall rate of drug release.

One of the advantages of using a Molecularly Imprinted Polymer as a drug delivery system, indeed, is that the release process consists of two different phases, namely binding-desorption and diffusion [39]. Firstly, the therapeutic agent desorbs from the selective binding cavities within the MIP, which act as reservoirs, becoming available and, then, it diffuses. This kind of two-stage release based on the combination of more than one mechanism allows one to overcome the drawbacks associated with Fickian diffusion. According to Fick’s law, indeed, the release of a drug from a matrix is non-linear over time due to the increase in diffusional length and/or the decrease in the releasing surface area [40]. Moreover, as the drug diffuses out, its concentration gradient decreases with time. Therefore, an effective drug delivery system must be designed to overcome Fick’s law in the aim to maintain the concentration of the therapeutic agent within the body at an optimal level and an effective approach to circumvent these drawbacks consists of the combination of more than one release mechanism. The developed MIP is able to combine diffusion and swelling mechanisms with binding-desorption and thus, it could represent a very interesting material for the design of a new delivery device.

## 4. Conclusions

The aim of the present research study was the preparation and characterization of a fluorescent Molecularly Imprinted Polymer selective for the anticancer drug Sunitinib (SUT) to be used for the development of a novel theranostic system that is able to integrate the drug controlled release ability of MIPs with Rhodamine 6G as a fluorescent marker.

Imprinted beads were prepared by precipitation polymerization and then, the obtained particles were functionalized with rhodamine 6G as a fluorescent marker by radical grafting using H_2_O_2_ and ascorbic acid as a redox pair.

Adsorption isotherms were investigated by batch binding studies, which confirmed the selective recognition properties of the synthesized MIP due to the presence of specific binding sites into the polymeric matrix. Moreover, the obtained binding data showed that SUT adsorption on MIP particles better fitted the Langmuir model and the kinetic study revealed that the adsorption process followed the pseudo-first order kinetic model for both the imprinted and non-imprinted materials.

Finally, the in vitro release studies highlighted the SUT controlled release behavior of MIP, which was well fitted with the Ritger-Peppas kinetic model.

The collected data confirmed that the synthesized fluorescent MIP represents a promising material for the development of a novel theranostic platform with a potential application in targeted cancer therapy. The auto-fluorescence of the developed imprinted polymeric particles, indeed, allows them not only to act as a drug carrier, but also to self-monitor the real-time MIP biodistribution and tumor accumulation via fluorescence imaging, leading to higher therapeutic accuracy in cancer treatment. The rhodamine incorporation into the polymeric matrix provided an integrated probe for both diagnostic purposes and guiding targeted therapy, combining fluorescence properties with highly selective recognition abilities.

## Figures and Tables

**Table 1:** Hydrodynamic mean diameter, Polydispersity Index (PI), and *ζ*-Potential for MIP and NIP particles.

Parameter	MIP	NIP
Hydrodynamic mean diameter (nm)	714.6 ± 9.7	709.2 ± 9.9
Polydispersity Index (PI)	0.305	0.297
*ζ*-Potential (mV)	−23.8 ± 0.3	−29.3 ± 0.4

**Table 2:** Percentages of bound Sunitinib (SUT) and Semaxanib (SEM) by imprinted (MIP) and non-imprinted (NIP) particles and *α* and *ε* values for different *C*_i_. Data are shown as means ± S.D.

*C*_i_ (mol/L)	Bound SUT (%)		Bound SEM (%)		*α* SUT	*α* SEM	*ɛ*
	MIP	NIP	MIP	NIP			
0.00001	47.2 ± 0.6	34.5 ± 0.5	12.5 ± 0.6	11.5 ± 0.4	1.37	1.09	3.77
0.00002	51.5 ± 0.7	42.5 ± 0.7	14.1 ± 0.6	11.8 ± 0.7	1.21	1.19	3.66
0.00004	56.3 ± 0.4	40.3 ± 0.3	11.8 ± 0.5	10.1 ± 0.6	1.40	1.16	4.79
0.00006	58.3 ± 0.5	41.7 ± 0.5	7.9 ± 0.8	6.6 ± 0.4	1.40	1.20	7.37
0.00008	66.9 ± 0.6	46.3 ± 0.4	26.9 ± 0.4	22.5 ± 0.8	1.45	1.19	2.49
0.0001	71.0 ± 0.7	47.9 ± 0.6	42.1 ± 0.5	37.9 ± 0.5	1.48	1.11	1.69
0.0002	75.0 ± 0.4	48.3 ± 0.6	50.2 ± 0.7	47.3 ± 0.5	1.55	1.06	1.49
0.0004	80.3 ± 0.8	50.0 ± 0.3	52.8 ± 0.5	51.3 ± 0.7	1.61	1.03	1.52
0.0006	77.5 ± 0.5	47.5 ± 0.8	50.8 ± 0.6	48.3 ± 0.3	1.63	1.05	1.52
0.0008	68.8 ± 0.3	46.3 ± 0.5	42.5 ± 0.4	40.1 ± 0.7	1.49	1.06	1.62

**Table 3:** *K*_a_, *B*_max_, and *R*^2^ values obtained by Scatchard analysis.

Polymer	High Affinity Sites			Low Affinity Sites		
	*K*_a_ (M^−1^)	*B*_max_ (mM/g)	*R* ^2^	*K*_a_ (M^−1^)	*B*_max_ (mM/g)	*R* ^2^
MIP	19,132	0.01	0.92	6,760	3.54 × 10^−3^	0.70
NIP	-	-	-	629.13	0.02	0.12

**Table 4:** *K*_L_, *Q*_max_ and *R*^2^, and *K*_F_, *m*, and *R*^2^ values obtained by Langmuir and Freundlich models, respectively.

Polymer	Langmuir Model			Freundlich Model		
	*K*_L_ (L/mol)	*Q*_max_ (mmol/g)	*R* ^2^	*K*_F_	*m*	*R* ^2^
MIP	10.29	1.74 × 10^−3^	0.98	0.25	1.19	0.93
NIP	4.40	2.51 × 10^−3^	0.98	0.03	1.07	0.99

**Table 5:** Kinetic fitting data for MIP and NIP.

Polymer	Pseudo-First Order			Pseudo-Second Order		
	*K*_1_	*Q*_e_	*R* ^2^	*K*_2_	*Q*_e_	*R* ^2^
MIP	0.36	8.61 × 10^−6^	0.97	4.27 × 10^3^	1.70 × 10^5^	0.77
NIP	0.30	5.39 × 10^−6^	0.97	1.58 × 10^3^	1.78 × 10^5^	0.63

**Table 6:** Drug loading content (DLC) and drug loading efficiency (DLE).

Drug Loading Content (DLC)		Drug Loading Efficiency (DLE)	
MIP	NIP	MIP	NIP
8.8 ± 0.1%	7.5 ± 0.3%	87.2 ± 0.2%	71.5 ± 0.4%

**Table 7:** Linear fitting of cumulative drug diffusion curves.

Polymer	Zero-Order Kinetic Model		First-Order Kinetic Model		Higuchi Kinetic Model		Ritger-Peppas Kinetic Model		
	*R* ^2^	*K*_0_	*R* ^2^	*K*_1_	*R* ^2^	*K*_H_	*R* ^2^	*K*_p_	*n*
MIP	0.6328	0.0297	0.7597	0.0659	0.8276	0.1884	0.9396	0.1589	0.8312
NIP	0.5062	0.0301	0.7196	0.1572	0.7283	0.2001	0.9865	0.2961	0.8178
